# Arterial and Venous Doppler Parameters in Fetal Growth Restriction: A Comparative Evaluation of Early- and Late-Onset Subtypes

**DOI:** 10.3390/diagnostics16101488

**Published:** 2026-05-14

**Authors:** Hale Ankara Aktas, Ilayda Gercik Arzik, Zubeyde Emiralioglu Cakir, Burak Bayraktar, Bahar Konuralp Atakul, Emre Bayram, Eyyup Suer Timur, Ibrahim Omeroglu, Atalay Ekin, Hakan Golbasi

**Affiliations:** 1 Department of Perinatology, Izmir City Hospital, Izmir 35010, Turkey; ilgercik@gmail.com (I.G.A.); baharkonuralp@gmail.com (B.K.A.); emrebayram029@gmail.com (E.B.); suertimur@gmail.com (E.S.T.); dribrahimomeroglu@gmail.com (I.O.); atalayekin@hotmail.com (A.E.); drhkngolbasi@gmail.com (H.G.); 2 Department of Perinatology, Buca Seyfi Demirsoy Education and Research Hospital, Izmir 35390, Turkey; zubeydeemiralioglu@hotmail.com; 3 Department of Perinatology, University of Health Sciences Ankara Etlik City Hospital, Ankara 06170, Turkey; drburakbayraktar@gmail.com

**Keywords:** fetal growth restriction, Doppler ultrasonography, umbilical artery, middle cerebral artery, ductus venosus, perinatal mortality

## Abstract

**Objective**: To evaluate and compare fetal arterial and venous Doppler parameters in early-onset (EO) and late-onset (LO) fetal growth restriction (FGR), and to assess their performance within the study cohort and their association with composite adverse neonatal outcome (CANO). **Methods**: This prospective observational cohort study included 184 singleton pregnancies between 24 and 37 weeks of gestation, comprising 91 FGR cases and 93 appropriate-for-gestational-age controls. FGR was defined according to Delphi consensus criteria and classified as EO-FGR (<32 weeks) or LO-FGR (≥32 weeks). All fetuses underwent standardized Doppler assessment of the umbilical artery (UA), middle cerebral artery (MCA), uterine artery (UtA), and ductus venosus (DV). The cerebroplacental ratio (CPR) was calculated. Multivariable logistic regression models were constructed separately for EO-FGR and LO-FGR. Classification performance was evaluated using receiver operating characteristic analysis. CANO was defined as at least one of the following: 5-min Apgar score <7, respiratory distress syndrome, neonatal intensive care unit admission, or preterm birth. **Results**: In both EO-FGR and LO-FGR, UA PI values were significantly higher, whereas MCA PI and CPR were significantly lower than in controls. CPR demonstrated the highest discriminative performance among arterial parameters in both subgroups. DV Doppler indices were not significantly different in EO-FGR. In LO-FGR, DV S-wave and v-wave velocities were independently associated with FGR. No significant associations were observed between Doppler parameters and CANO in subgroup analyses. **Conclusions**: Arterial Doppler parameters, particularly CPR, showed consistent alterations in both EO- and LO-FGR. The contribution of venous Doppler parameters differed according to clinical subtype, with additional value observed in LO-FGR.

## 1. Introduction

Fetal growth restriction (FGR) affects approximately 5–10% of pregnancies and remains one of the principal determinants of perinatal morbidity and mortality [1,2]. Contemporary definitions extend beyond fetal biometric thresholds and incorporate evidence of placental dysfunction and associated fetal hemodynamic adaptations, rather than relying solely on estimated fetal weight (EFW) or abdominal circumference (AC) below specific percentile cut-offs [3,4]. Increased placental vascular resistance and impaired uteroplacental perfusion lead to compensatory circulatory responses in the fetus, which can be quantified using Doppler velocimetry [4].

The distinction between early-onset (EO-FGR) and late-onset (LO-FGR) subtypes is essential for clinical management and prognostic stratification. EO-FGR, typically diagnosed before 32 weeks of gestation, is commonly associated with severe placental insufficiency. In this subgroup, elevated umbilical artery (UA) impedance is detected early, followed by middle cerebral artery (MCA) vasodilatation and a reduction in the cerebroplacental ratio (CPR), reflecting a progressive pattern of hemodynamic redistribution [5,6]. In advanced stages, venous involvement emerges, and alterations in ductus venosus (DV) flow patterns are regarded as late markers of fetal cardiovascular decompensation [6]. Longitudinal studies have demonstrated that abnormalities in UA and MCA Doppler parameters often precede delivery by several weeks, whereas DV Doppler deterioration generally manifests later in the course of the disease [7,8,9]. In contrast, LO-FGR is characterized by milder and more heterogeneous placental dysfunction. UA Doppler indices frequently remain within normal limits, while cerebral redistribution and reductions in CPR are more prominent findings [6,10,11].

In normal fetal circulation, the DV functions as a physiological shunt, directing a substantial proportion of oxygenated blood from the umbilical vein toward the heart. Its flow characteristics are sensitive to changes in cardiac preload and central venous pressure [12]. In EO-FGR, DV Doppler abnormalities have been described as indicators of advanced cardiovascular compromise, with absent or reversed a-wave strongly associated with perinatal mortality [12,13,14]. In LO-FGR, DV flow patterns are generally preserved, although subtle alterations have been reported in selected cohorts [6,11]. Despite this pathophysiological framework, studies simultaneously comparing arterial and venous Doppler parameters across EO-FGR and LO-FGR subtypes, and evaluating their associations with perinatal outcomes at the subtype level, remain limited [15,16,17].

The present study aimed to compare fetal arterial (UA, MCA, CPR) and venous (DV) Doppler parameters in pregnancies diagnosed with FGR between 24 and 37 weeks of gestation with those of fetuses appropriate for gestational age (AGA). In addition, we sought to evaluate the classification performance of these parameters in EO-FGR and LO-FGR subgroups and to assess their associations with composite adverse neonatal outcome (CANO).

## 2. Materials and Methods

### 2.1. Study Design and Setting

This prospective observational cohort study was conducted at the Department of Perinatology, Izmir City Hospital, Izmir, Turkey, a tertiary perinatology referral center, between November 2024 and November 2025. The study cohort consisted exclusively of participants from a Turkish population. The study protocol was approved by the local ethics committee (approval number: 2024/212) and conducted in accordance with the ethical principles of the Declaration of Helsinki. The study was prospectively registered on ClinicalTrials.gov (Identifier: NCT07190157). All participants were informed about the study and written informed consent was obtained.

### 2.2. Study Population

Consecutive eligible singleton pregnancies between 24 and 37 weeks of gestation were recruited during the study period. A total of 184 singleton pregnancies were included, including 91 pregnancies complicated by FGR and 93 healthy control pregnancies. FGR was defined according to the Delphi consensus criteria, which integrate fetal biometric measurements with Doppler findings [3]. In EO-FGR, defined as diagnosis before 32 weeks of gestation, FGR was diagnosed in the presence of an EFW below the 3rd percentile, or an EFW below the 10th percentile in association with abnormal placental Doppler findings, defined as a pulsatility index (PI) above the 95th percentile in the UA and/or uterine artery (UtA) for gestational age. In LO-FGR (≥32 weeks of gestation), FGR was defined as an EFW below the 3rd percentile, or an EFW below the 10th percentile combined with at least two of the following criteria: AC below the 10th percentile, UA PI above the 95th percentile, or CPR below the 5th percentile for gestational age. EFW and AC percentiles were determined according to the Hadlock reference charts [18].

All fetuses underwent standardized Doppler assessment, including measurement of UA PI, MCA PI, UtA PI, calculation of the CPR, and evaluation of DV flow parameters. FGR cases were further stratified into EO-FGR and LO-FGR subgroups and analyzed separately with respect to Doppler findings as well as perinatal and neonatal outcomes. The study was not designed to evaluate longitudinal Doppler progression between trimesters. The control group consisted of consecutively recruited singleton pregnancies evaluated during the same study period at the same center and classified as AGA, defined by AC and EFW measurements between the 10th and 90th percentiles. Control pregnancies met the same exclusion criteria as the FGR group.

Multiple pregnancies, cases with major fetal structural or chromosomal anomalies, pregnancies complicated by maternal systemic diseases, maternal age below 18 years, presence of perinatal infection, and pregnancies conceived by in vitro fertilization (IVF) were excluded from the study. Maternal systemic diseases included hypertensive disorders of pregnancy, pregestational diabetes mellitus, renal disease, autoimmune disorders, and other clinically significant maternal medical conditions that could affect placentation or fetal growth. IVF pregnancies were excluded to minimize potential confounding related to assisted reproductive technology, which may independently influence placentation, fetal growth patterns, and Doppler findings. Maternal age, parity, obstetric history, and other relevant maternal clinical characteristics were recorded for all participants. Maternal body mass index (BMI) was calculated at the time of ultrasound assessment using weight and height recorded during the study visit. Gestational age was determined based on the last menstrual period and was confirmed in all cases by first-trimester crown–rump length (CRL) measurements. Clinical follow-up and management of pregnancies complicated by FGR were carried out in accordance with the guidelines of the International Society of Ultrasound in Obstetrics and Gynecology (ISUOG) [5].

### 2.3. Ultrasound Examinations

Doppler evaluation of the UA, MCA, UtA, and DV was conducted in accordance with standard Doppler measurement principles. All ultrasonographic examinations were performed using a Voluson E8 ultrasound system (GE Healthcare, Zipf, Austria) equipped with a 2–9 MHz convex transducer.

Standard fetal biometric measurements, including biparietal diameter (BPD), head circumference (HC), AC, and femur length (FL), were obtained. In addition to biometric assessment, Doppler examinations of the UA, MCA, UtA, and DV were performed. Doppler measurements were obtained during periods of minimal fetal activity and when the fetal heart rate was within normal limits.

For UA and MCA Doppler assessments, the PI was calculated and peak systolic velocity (PSV) was additionally measured for the MCA. Uterine artery Doppler evaluation included PI measurements obtained from both uterine arteries. The CPR was calculated as the ratio of MCA PI to UA PI.

**Figure 1:**
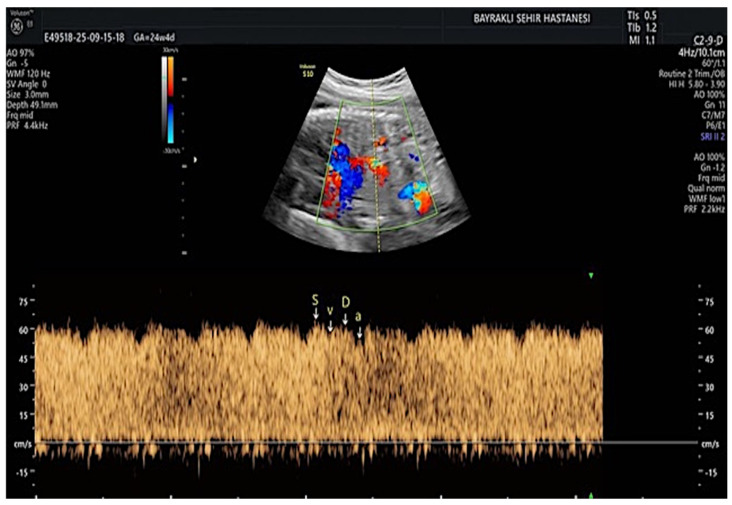
Spectral Doppler waveform of the ductus venosus demonstrating S−, D−, v−, and a−wave velocities.

DV Doppler assessment was performed in accordance with established technical principles as described in the literature [19]. The DV was identified using color Doppler imaging in a sagittal or oblique plane of the fetal abdomen, demonstrating the characteristic aliasing pattern caused by high-velocity flow at the vessel inlet. The sample volume was set between 0.5 and 1.0 mm and positioned at the isthmic region and adjacent proximal segment where accelerated jet flow was visualized. A pulsed Doppler was then used to record blood flow velocity waveforms at the inlet of the DV. During measurements, care was taken to keep the insonation angle as low as possible (<30°) and to ensure clear waveform morphology. Only Doppler recordings of adequate technical quality containing at least three consecutive cardiac cycles were included in the analysis (Figure 1). Pulsatility indices and velocity-based Doppler parameters derived from DV waveforms were analyzed. All ultrasonographic and Doppler measurements were performed by a perinatology specialist experienced in obstetric Doppler assessment.

### 2.4. Outcome Measures

The CANO was defined as the presence of at least one of the following: a 5-min Apgar score below 7, development of respiratory distress syndrome (RDS), admission to the neonatal intensive care unit (NICU), or preterm birth.

### 2.5. Statistical Analysis

All statistical analyses were performed using IBM SPSS Statistics (Version 26.0, IBM Corp., Armonk, NY, USA). Variables were expressed as mean ± standard deviation (SD) and compared using the independent samples *t*-test. Categorical variables were presented as number (%) and compared using the Chi-square test or Fisher’s exact test when required. Although the study population comprised three clinical categories (EO-FGR, LO-FGR, and controls), comparisons were performed as two separate binary analyses: (1) EO-FGR versus its corresponding control group and (2) LO-FGR versus its corresponding control group. The study was not designed as a matched case-control analysis; baseline maternal characteristics were compared between groups, and clinically relevant covariates were adjusted for in multivariable models.

To identify independent predictors of EO-FGR and LO-FGR, separate multivariate binary logistic regression models were constructed. Maternal age, BMI, parity, and gestational age at measurement were included as covariates in all models. To avoid multicollinearity among arterial Doppler indices, UA PI, MCA PI, and CPR were not entered simultaneously in the same model. Instead, three separate arterial models were constructed: Model A: UA PI; Model B: MCA PI; and Model C: CPR. For LO-FGR, an additional venous Doppler–based model (Model D) was constructed incorporating DV S, DV D, DV v, and DV TAmax. Individual DV parameters were not modeled separately to reduce the risk of multiple testing bias and improve clinical interpretability. Adjusted odds ratios (ORs) with 95% confidence intervals (CIs) were calculated. The classification performance of each multivariate model was evaluated using receiver operating characteristic (ROC) curve analysis, and the area under the curve (AUC) was calculated. A two-sided *p*-value < 0.05 was considered statistically significant.

## 3. Results

A total of 184 singleton pregnancies were included in the study, comprising 91 cases diagnosed with FGR and 93 control cases. Maternal age was comparable between groups, whereas maternal BMI was higher in the control group. Pregnancies complicated by FGR delivered earlier and had lower birth weights than controls. Neonatal condition at birth was poorer in the FGR group, with lower Apgar scores and higher rates of RDS, NICU admission, and CANO. Detailed demographic, maternal, obstetric, and neonatal characteristics of the study groups are presented in Table 1.

When fetal Doppler measurements were compared across EO-FGR, LO-FGR, and control groups, UtA PI values were similar across groups. In both EO-FGR and LO-FGR, arterial Doppler findings showed a similar pattern with higher UA PI and lower MCA PI and CPR values compared with controls. DV Doppler indices showed no significant differences in EO-FGR. In contrast, several velocity-based DV parameters, including S-wave, D-wave, v-wave, and TAmax, were higher in the LO-FGR group than in controls. Comparative fetal Doppler findings for EO-FGR and LO-FGR groups are summarized in Table 2.

Multivariable logistic regression analyses for EO-FGR are presented in Table 3. Across separate models, higher UA PI, lower MCA PI, and lower CPR were independently associated with EO-FGR. Maternal BMI and parity also remained significant in selected models.

Multivariable logistic regression analyses for LO-FGR were presented in Table 4. Higher UA PI, lower MCA PI, lower CPR, and selected velocity-based DV parameters were independently associated with LO-FGR. Maternal BMI remained significant across models, and parity was significant in the venous Doppler model.

Comparisons according to CANO status were associated with less favorable obstetric and neonatal characteristics among affected pregnancies, including earlier delivery, higher rates of preterm birth and cesarean section, and lower birth weight. Detailed findings according to CANO status were presented in Table 5.

Within the EO-FGR group, fetal Doppler parameters were similar between pregnancies with and without CANO, with no statistically significant differences observed. Likewise, within the LO-FGR group, no significant differences were detected according to CANO status. Although UA PI values tended to be higher in LO-FGR cases with CANO, this did not reach statistical significance. Detailed fetal Doppler findings according to CANO status in EO-FGR and LO-FGR groups are presented in Table 6.

**Figure 2:**
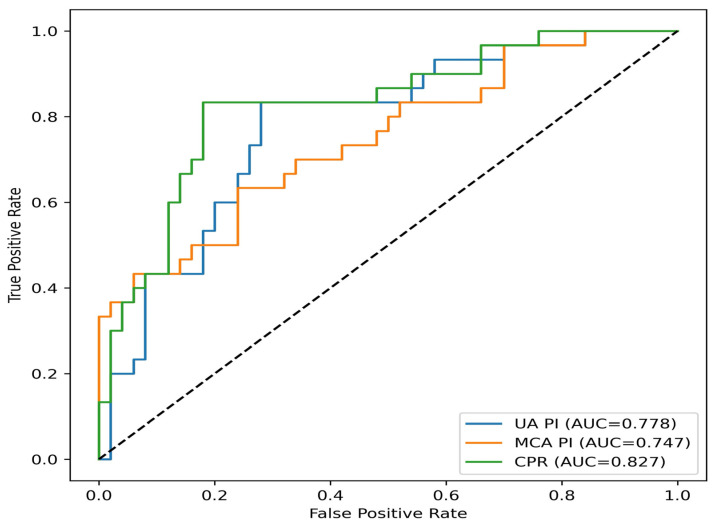
Receiver operating characteristic (ROC) curves comparing the classification performance of arterial Doppler-based models (umbilical artery PI, middle cerebral artery PI, cerebroplacental ratio) for differentiation of early-onset fetal growth restriction (EO-FGR) cases from controls within the study cohort.

**Figure 3:**
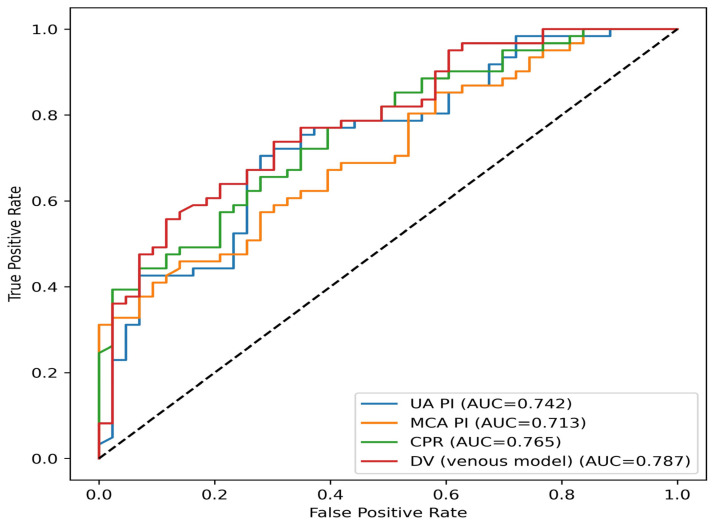
Receiver operating characteristic (ROC) curves comparing the classification performance of arterial Doppler-based models (umbilical artery PI, middle cerebral artery PI, cerebroplacental ratio) and a venous Doppler-based model for differentiation of late-onset fetal growth restriction (LO-FGR) cases from controls within the study cohort.

Receiver operating characteristic (ROC) curve analyses demonstrated that among models constructed for EO-FGR, the CPR-based model exhibited the highest classification performance within the cohort (AUC = 0.827). Models based on UA PI (AUC = 0.778) and MCA PI (AUC = 0.747) showed lower classification performance compared with the CPR-based model (Figure 2). In the LO-FGR group, CPR again demonstrated the highest classification performance among arterial Doppler-based models (AUC = 0.765), followed by UA PI (AUC = 0.742) and MCA PI (AUC = 0.713). The venous Doppler model incorporating DV parameters showed better performance than the arterial models (AUC = 0.787) (Figure 3).

## 4. Discussion

In this study, fetal Doppler parameters were systematically assessed in EO-FGR and LO-FGR and compared with those of AGA fetuses. The principal findings demonstrated that UA and MCA Doppler indices exhibited similar directional changes in both FGR subgroups compared with AGA fetuses, and that the CPR emerged as a common arterial Doppler parameter reflecting uteroplacental insufficiency across both phenotypes. In contrast, the relative contribution of venous Doppler parameters differed between subtypes; whereas no clear additional association was observed in EO-FGR, several velocity-based DV indices were significantly altered in LO-FGR. These results suggest that arterial Doppler assessment provides a common baseline for both groups in FGR monitoring, whereas venous Doppler may provide complementary information in LO-FGR.

FGR is generally considered to be associated with uteroplacental circulatory insufficiency in many cases [20]. In this process, increased placental vascular resistance and the accompanying fetal circulatory adaptations lead to measurable changes in arterial Doppler parameters. In the literature, increased UA PI and decreased MCA PI have been described as early indicators of the adaptive response to uteroplacental insufficiency and fetal hypoxemia, and these changes have been reported to occur with similar patterns regardless of the time of clinical onset of FGR [5,20,21]. Madazlı et al. showed that UA, MCA and CPR Doppler patterns in EO-FGR and LO-FGR cases were associated with the severity of FGR and perinatal outcomes, although partially overlapping findings could be observed between groups [22]. Stampalija et al. reported that changes in MCA PI and the umbilicocerebral ratio in pregnancies at risk of FGR during the late preterm period reflect cerebral redistribution secondary to uteroplacental insufficiency, but that these alterations do not provide clear discrimination in all cases [23]. CPR is an arterial Doppler index based on the combined assessment of MCA PI, which reflects fetal cerebral perfusion, and UA PI, which provides information on placental resistance [24]. The literature reports that CPR may be more sensitive than the isolated use of UA or MCA in demonstrating hemodynamic involvement secondary to uteroplacental insufficiency and may contribute to the prediction of perinatal risk [24,25]. Consistent with the arterial Doppler changes reported in the literature, the findings of our study showed that increased UA PI and decreased MCA PI and CPR were evident in both EO-FGR and LO-FGR cases, and that CPR was the arterial parameter showing the strongest differentiation between study groups.

In FGR, the course of Doppler findings reflects different hemodynamic adaptations depending on gestational age at diagnosis. In EO-FGR, increased UA PI is detected in most cases at an early stage due to placental insufficiency, whereas changes in the cerebral circulation and abnormalities in DV Doppler generally appear at more advanced gestational weeks [13]. In LO-FGR, UA PI often remains within the normal range, which leads to MCA vasodilation and a decrease in CPR, which become more prominent indicators of fetal adaptation [6]. These differences suggest that EO-FGR and LO-FGR should be considered as distinct pathophysiological processes not only in terms of clinical severity but also with respect to the diagnostic and prognostic values of Doppler parameters. In this context, when our findings are considered in the context of the literature, the similar directional changes in UA and MCA Doppler parameters in both EO-FGR and LO-FGR indicate that arterial Doppler impairment constitutes a common hemodynamic response in FGR. However, the similar directional arterial Doppler response observed in our study does not provide clear discrimination with respect to the time of clinical onset.

Late-onset FGR should also be considered a clinically distinct phenotype, typically presenting closer to term with subtler placental dysfunction and less pronounced venous compromise than EO-FGR. In these pregnancies, cerebral redistribution and reductions in CPR may be more informative than severe UA or DV deterioration [6,11,17]. Consistent with this concept, our LO-FGR subgroup demonstrated clear arterial Doppler changes, whereas conventional DV pulsatility indices remained largely preserved [11,17]. These findings support the importance of careful arterial Doppler surveillance in late-onset disease, while venous Doppler abnormalities may be limited or occur later in the disease course [16,17].

In the hemodynamic adaptation process in FGR, it is generally accepted that the venous system, particularly the DV, is affected later than the arterial circulation [7]. In EO-FGR, deterioration in DV Doppler is classically considered a late indicator of advanced placental insufficiency and cardiac decompensation [6]. In EO-FGR, with the progression of severe placental insufficiency, DV PI has been shown to increase gradually. In cases with impaired flow particularly in the final weeks, a reduction in a-wave amplitude and even loss or reversal of the a-wave in advanced stages have been reported as late findings associated with fetal acidosis and perinatal mortality [12,13,14]. It has been reported that in EO-FGR, DV becomes abnormal generally in the days immediately preceding delivery compared with UA and MCA Doppler, and for this reason it is used as one of the late indicators in determining the timing of delivery [6]. In contrast, in LO-FGR, because uteroplacental insufficiency is milder, most studies have reported that UA PI may remain within normal limits, hemodynamic deterioration primarily follows cerebral vasodilation and a decrease in CPR, and DV flow is generally preserved or limited to a mild increase in PI [11,16]. When our findings are considered in the context of the literature, the significantly higher DV S, DV D, DV v, and DV TAmax values observed in the LO-FGR group may suggest subtle alterations in venous hemodynamics. However, classical venous Doppler indices such as PIV, PVIV, PLI, and a-wave-related ratios were not significantly different between groups. In addition, velocity-based parameters may be more susceptible to insonation angle, fetal activity, preload conditions, and physiologic variation than conventional venous indices. As these parameters were analyzed as absolute velocity values rather than gestational age-standardized centiles or multiples of the median, residual effects related to gestational age or fetal size cannot be fully excluded. Although these findings do not directly indicate increased cardiovascular loading, they may reflect subtle physiologic or hemodynamic variation in LO-FGR cases.

In the literature, particularly in EO-FGR, gestational age is the strongest determinant of perinatal mortality and severe neonatal morbidity, while advanced Doppler abnormalities contribute to risk stratification [22,26]. In addition, Doppler findings in EO-FGR have clinical significance not only in antenatal surveillance but also for postnatal neurodevelopmental outcomes. In the TRUFFLE randomized trial conducted by Lees et al., in EO-FGR cases between 26 and 32 weeks of gestation, timing of delivery based on late changes in the DV waveform, specifically loss or reversal of the a-wave, compared with computerized cardiotocography short-term variation. This approach was associated with increased survival without neurological impairment at 2 years, despite similar perinatal mortality rates [26]. Although DV Doppler is described in the literature as a late indicator of cardiovascular decompensation, particularly in severe and advanced EO-FGR, no significant association was observed between DV parameters and perinatal outcomes in the EO-FGR subgroup in our study. This finding may be explained by the possibility that most cases were delivered before reaching the stage of venous insufficiency and DV abnormalities generally occur only in the most severe EO-FGR phenotypes.

In our cohort, gestational age at delivery was lower in the FGR groups than in AGA controls. However, the mean gestational age at delivery in our FGR cohort appears relatively higher than expected, which may be related to the proportion of LO-FGR cases. This likely reflects center-specific case characteristics. This finding likely reflects closer antenatal surveillance and a lower threshold for delivery in pregnancies considered at increased risk of placental insufficiency or fetal compromise. In contrast, pregnancies in the control group were generally managed expectantly until spontaneous labor or routine term delivery in the absence of maternal or fetal indications for earlier birth. Therefore, the observed difference in delivery timing likely reflects differences in obstetric management according to the level of pregnancy risk [5].

The cesarean delivery rate was high in both groups and was higher in the control than in the FGR group. This finding may be related to a higher frequency of previous cesarean delivery (scarred uterus) in the control group. In addition, our hospital is a tertiary referral center, where cesarean delivery rates may be influenced by referral patterns, physician preference, and cautious obstetric management. National data from Turkey have also reported relatively high cesarean section rates compared with many countries [27,28].

Although the control group consisted of AGA pregnancies, NICU admission was observed in a small proportion of newborns. This may be related to transient respiratory adaptation issues, short-term observation requirements, neonatal hypoglycemia, jaundice, or precautionary monitoring in a tertiary care setting. In addition, the relatively high cesarean delivery rate in the control group may have contributed to increased short-term respiratory morbidity requiring neonatal observation [29,30].

One of the main strengths of this study is its prospective observational design and the evaluation of EO-FGR and LO-FGR cases as well-defined subgroups. The systematic assessment of arterial and venous Doppler parameters within the same cohort, performed in accordance with standard measurement principles by an experienced perinatology specialist, increases the consistency of the findings. However, the study has several limitations. The reliance on single time-point Doppler measurements and the absence of longitudinal follow-up may have limited the evaluation of the temporal course of venous Doppler deterioration in particular. In addition, DV velocity parameters were evaluated as absolute values rather than gestational age-standardized centiles or multiples of the median, which may limit physiologic interpretation of these findings. Although the sample size was adequate for EO-FGR and LO-FGR subgroup analyses, the relatively limited number of cases with advanced venous Doppler abnormalities and severe neonatal morbidity should be considered when interpreting these findings, as it may have limited the ability to demonstrate statistically significant associations between DV parameters and neonatal outcomes. Larger prospective studies may help confirm these observations. In addition, subgroup analyses according to CANO status involved relatively small sample sizes, which may have reduced statistical power and increased the possibility of type II error. Therefore, non-significant findings should be interpreted cautiously. The absence of postnatal long-term neurodevelopmental follow-up data limits the assessment of the impact of Doppler findings on long-term clinical outcomes.

## 5. Conclusions

In conclusion, arterial Doppler parameters and CPR showed consistent directional changes in both EO-FGR and LO-FGR subgroups and remain important components in the surveillance of FGR. In contrast, the contribution of venous Doppler parameters differed according to clinical subtype of FGR and selected DV velocity measurements may provide complementary information, particularly in LO-FGR. While gestational age remains a major determinant of adverse neonatal outcomes, fetal Doppler assessment remains an important complementary tool in understanding fetal circulatory adaptations. These results support the interpretation of Doppler parameters in FGR surveillance in conjunction with gestational age and the clinical context.

## Figures and Tables

**Table 1:** Maternal, obstetric, and neonatal characteristics of the study groups.

	FGR (*n* = 91)	Control (*n* = 93)	*p*-Value
**Maternal age (years) (mean ± SD)**	27.8 ± 5.1	28.4 ± 5.2	0.437
**BMI at assessment (kg/m^2^) (mean ± SD)**	27.1 ± 4.0	29.0 ± 4.4	0.003
**Parity (*n*, %)**			0.007
**Nulliparous**	57 (62.6%)	40 (43%)	
**Multiparous**	34 (37.4%)	53 (57%)	
**Gestational age at study measurement (weeks) (mean ± SD)**	34.3 ± 2.3	33.3 ± 2.6	0.121
**Gestational age at delivery (weeks) (mean ± SD)**	37.1 ± 1.4	38.3 ± 1.6	<0.001
**Preterm birth (<37 weeks) (*n*, %)**	13 (14.3%)	9 (9.6%)	0.335
**Cesarean section (*n*, %)**	48 (52.7%)	66 (71%)	0.010
**Birth weight (g) (mean ± SD)**	2418 ± 457	3186 ± 456	<0.001
**1st-minute Apgar score (mean ± SD)**	7.65 ± 0.52	7.85 ± 0.44	0.007
**5th-minute Apgar score (mean ± SD)**	8.79 ± 0.46	8.97 ± 0.24	0.002
**5th-minute Apgar score < 7 (*n*, %)**	9 (9.9%)	1 (1.1%)	0.008
**RDS (*n*, %)**	23 (25.3%)	8 (8.6%)	0.005
**NICU admission (*n*, %)**	23 (25.3%)	8 (8.6%)	0.005
**Composite adverse neonatal outcome** * **(*n*, %)**	26 (28.6%)	8 (8.6%)	0.001
**Perinatal mortality (*n*, %)**	0 (0%)	0 (0%)	N/A

BMI: body mass index; RDS: respiratory distress syndrome; NICU: neonatal intensive care unit; SD: standard deviation. * Composite adverse neonatal outcome (CANO) was defined as at least one of the following: 5-min Apgar score < 7, RDS, NICU admission, or preterm birth.

**Table 2:** Comparison of fetal Doppler measurements between EO-FGR, LO-FGR, and control groups.

	EO-FGR (*n* = 40)	Control (*n* = 45)	*p*-Value	LO-FGR (*n* = 51)	Control (*n* = 48)	*p*-Value
**Right uterine artery PI (mean ± SD)**	0.88 ± 0.36	0.81 ± 0.28	0.326	0.80 ± 0.35	0.76 ± 0.29	0.483
**Left uterine artery PI (mean ± SD)**	0.96 ± 0.44	0.81 ± 0.30	0.103	0.80 ± 0.29	0.76 ± 0.27	0.481
**UA PI (mean ± SD)**	1.04 ± 0.16	0.95 ± 0.14	0.013	0.95 ± 0.18	0.85 ± 0.17	0.005
**MCA PI (mean ± SD)**	1.80 ± 0.34	2.03 ± 0.33	0.004	1.62 ± 0.31	1.76 ± 0.33	0.030
**MCA PSV (cm/s) (mean ± SD)**	46.96 ± 8.94	49.18 ± 8.83	0.282	53.45 ± 10.36	56.77 ± 8.52	0.087
**DV PLI (mean ± SD)**	0.52 ± 0.18	0.46 ± 0.13	0.119	0.44 ± 0.13	0.44 ± 0.16	0.966
**DV PVIV (mean ± SD)**	0.65 ± 0.39	0.58 ± 0.27	0.329	0.54 ± 0.20	0.57 ± 0.33	0.602
**DV PIV (mean ± SD)**	0.66 ± 0.27	0.57 ± 0.20	0.144	0.55 ± 0.20	0.56 ± 0.27	0.809
**DV D/a (mean ± SD)**	2.31 ± 2.52	1.61 ± 0.42	0.145	1.61 ± 0.40	1.66 ± 0.47	0.621
**DV v/a (mean ± SD)**	2.02 ± 2.30	1.48 ± 0.33	0.211	1.47 ± 0.27	1.45 ± 0.39	0.750
**DV S/a (mean ± SD)**	2.73 ± 1.34	1.99 ± 0.56	0.099	1.93 ± 0.57	2.03 ± 0.83	0.508
**DV S/v (mean ± SD)**	1.36 ± 0.29	1.31 ± 0.20	0.414	1.30 ± 0.19	1.34 ± 0.37	0.437
**DV S/D (mean ± SD)**	1.21 ± 0.36	1.22 ± 0.28	0.849	1.19 ± 0.17	1.19 ± 0.34	0.918
**DV v/D (mean ± SD)**	0.88 ± 0.13	0.93 ± 0.14	0.201	0.93 ± 0.14	0.89 ± 0.15	0.218
**DV S (mean ± SD)**	59.56 ± 18.56	53.68 ± 18.63	0.175	57.54 ± 20.29	45.95 ± 17.46	0.003
**DV D (mean ± SD)**	52.49 ± 21.08	45.89 ± 18.93	0.152	48.86 ± 17.45	41.35 ± 17.74	0.034
**DV a (mean ± SD)**	29.73 ± 15.45	30.15 ± 14.29	0.903	31.42 ± 12.55	26.68 ± 12.88	0.063
**DV v (mean ± SD)**	45.51 ± 16.77	41.84 ± 16.54	0.342	44.75 ± 15.23	35.77 ± 13.44	0.002
**DV TAmax (mean ± SD)**	48.87 ± 17.03	44.74 ± 16.96	0.296	47.75 ± 16.16	39.54 ± 14.66	0.009
**CPR (mean ± SD)**	1.76 ± 0.44	2.19 ± 0.48	<0.001	1.75 ± 0.47	2.13 ± 0.59	0.001

PI: pulsatility index; UA: umbilical artery; S/D: systolic/diastolic; MCA: middle cerebral artery; PSV: peak systolic velocity; DV: ductus venosus; PLI: preload index; PVIV: peak velocity index for veins; PIV: pulsatility index for veins; S: systolic peak velocity; D: diastolic peak velocity; a: peak velocity during atrial contraction; v: peak velocity during isovolumetric ventricular relaxation; TAmax: time-averaged maximum velocity; CPR: cerebroplacental ratio; SD: standard deviation.

**Table 3:** Multivariate logistic regression analysis of independent predictors of EO-FGR.

	OR	95% CI	*p*-Value
***Model A***			
**Maternal age (years)**	1.11	0.99–1.24	0.081
**BMI at assessment (kg/m^2^)**	0.86	0.76–0.98	0.022
**Parity**	0.45	0.22–0.92	0.028
**Gestational age at measurement (weeks)**	1.12	0.84–1.49	0.435
**UA PI**	231.66	5.83–9199.45	0.003
***Model B***			
**Maternal age (years)**	1.08	0.97–1.21	0.125
**BMI at assessment (kg/m^2^)**	0.90	0.80–1.01	0.091
**Parity**	0.49	0.24–1.00	0.050
**Gestational age at measurement (weeks)**	1.07	0.81–1.41	0.631
**MCA PI**	0.01	0.01–0.57	0.010
***Model C***			
**Maternal age (years)**	1.12	0.99–1.27	0.069
**BMI at assessment (kg/m^2^)**	0.86	0.76–0.98	0.028
**Parity**	0.44	0.21–0.95	0.036
**Gestational age at measurement (weeks)**	1.22	0.88–1.68	0.226
**CPR**	0.06	0.01–0.28	<0.001

OR: odds ratio; CI: confidence interval; BMI: body mass index; UA: umbilical artery; PI: pulsatility index; MCA: middle cerebral artery; CPR: cerebroplacental ratio.

**Table 4:** Multivariate logistic regression analysis of independent predictors of LO-FGR.

	OR	95% CI	*p*-Value
***Model A***			
**Maternal age (years)**	0.99	0.89–1.09	0.830
**BMI at assessment (kg/m^2^)**	0.86	0.76–0.97	0.011
**Parity**	0.71	0.44–1.15	0.170
**Gestational age at measurement (weeks)**	1.27	0.76–2.15	0.360
**UA PI**	35.81	2.63–486.92	0.007
***Model B***			
**Maternal age (years)**	0.99	0.89–1.00	0.857
**BMI at assessment (kg/m^2^)**	0.84	0.74–0.95	0.006
**Parity**	0.74	0.46–1.20	0.225
**Gestational age at measurement (weeks)**	1.25	0.73–2.05	0.441
**MCA PI**	0.20	0.05–0.86	0.031
***Model C***			
**Maternal age (years)**	0.98	0.88–1.09	0.68
**BMI at assessment (kg/m^2^)**	0.84	0.74–0.95	0.007
**Parity**	0.77	0.47–1.26	0.300
**Gestational age at measurement (weeks)**	1.13	0.66–1.95	0.650
**CPR**	0.21	0.08–0.55	0.001
***Model D***			
**Maternal age (years)**	1.04	0.94–1.15	0.480
**BMI at assessment (kg/m^2^)**	0.83	0.72–0.96	0.011
**Parity**	0.59	0.36–0.97	0.037
**Gestational age at measurement (weeks)**	1.02	0.58–1.82	0.930
**DV S**	1.15	1.00–1.32	0.044
**DV D**	0.98	0.88–1.09	0.710
**DV v**	1.20	1.02–1.41	0.025
**DV TAmax**	0.78	0.58–1.05	0.096

OR: odds ratio; CI: confidence interval; BMI: body mass index; UA: umbilical artery; PI: pulsatility index; CPR: cerebroplacental ratio; DV: ductus venosus; S: systolic peak velocity; D: diastolic peak velocity; v: peak velocity during isovolumetric ventricular relaxation; TAmax: time-averaged maximum velocity.

**Table 5:** Comparison of maternal characteristics, obstetric features, and fetal Doppler measurements between neonates with and without composite adverse neonatal outcome (CANO).

	with CANO (*n* = 34)	Without CANO (*n* = 150)	*p*-Value
**Maternal age (years) (mean ± SD)**	28.8 ± 5.9	28.0 ± 5.0	0.428
**BMI at assessment (kg/m^2^) (mean ± SD)**	27.8 ± 5.1	28.1 ± 4.1	0.771
**Parity (*n*, %)**			0.976
**Nulliparous**	18 (52.9%)	79 (52.6%)	
**Multiparous**	16 (47.1%)	71 (47.4%)	
**Gestational age at measurement (weeks) (mean ± SD)**	33.2 ± 2.4	34.2 ± 2.3	0.105
**Gestational age at delivery (weeks) (mean ± SD)**	36.2 ± 1.9	38.1 ± 1.2	<0.001
**Preterm birth (<37 weeks) (*n*, %)**	16 (47%)	6 (4%)	<0.001
**Cesarean section (*n*, %)**	29 (85.3%)	85 (56.6%)	0.002
**Birth weight (g) (mean ± SD)**	2084 ± 424	2959 ± 501	<0.001

CANO: composite adverse neonatal outcome; BMI: body mass index; SD: standard deviation.

**Table 6:** Comparison of fetal Doppler measurements between EO-FGR and LO-FGR groups with and without CANO.

	EO-FGR (*n* = 40)			LO-FGR (*n* = 51)		
	with CANO (*n* = 16)	Without CANO (*n* = 24)	*p*-Value	with CANO (*n* = 10)	Without CANO (*n* = 41)	*p*-Value
**Right uterine artery PI (mean ± SD)**	0.86 ± 0.35	0.90 ± 0.39	0.777	0.86 ± 0.33	0.79 ± 0.36	0.575
**Left uterine artery PI (mean ± SD)**	1.06 ± 0.53	0.85 ± 0.30	0.189	0.87 ± 0.27	0.78 ± 0.29	0.367
**UA PI (mean ± SD)**	1.06 ± 0.14	1.02 ± 0.18	0.474	1.07 ± 0.19	0.93 ± 0.17	0.058
**MCA PI (mean ± SD)**	1.76 ± 0.37	1.85 ± 0.31	0.455	1.62 ± 0.39	1.62 ± 0.30	0.994
**MCA PSV (cm/s) (mean ± SD)**	48.12 ± 8.91	45.64 ± 9.13	0.459	51.81 ± 10.26	53.77 ± 10.46	0.590
**DV PLI (mean ± SD)**	0.49 ± 0.16	0.55 ± 0.20	0.407	0.47 ± 0.16	0.44 ± 0.12	0.562
**DV PVIV (mean ± SD)**	0.57 ± 0.20	0.75 ± 0.53	0.250	0.63 ± 0.32	0.52 ± 0.17	0.305
**DV PIV (mean ± SD)**	0.62 ± 0.24	0.70 ± 0.30	0.467	0.60 ± 0.26	0.54 ± 0.19	0.386
**DV D/a (mean ± SD)**	1.86 ± 0.76	2.83 ± 3.60	0.302	1.59 ± 0.37	1.62 ± 0.41	0.854
**DV v/a (mean ± SD)**	1.53 ± 0.46	2.58 ± 3.30	0.218	1.49 ± 0.30	1.47 ± 0.26	0.791
**DV S/a (mean ± SD)**	2.24 ± 0.91	3.28 ± 3.26	0.232	2.08 ± 0.73	1.91 ± 0.53	0.397
**DV S/v (mean ± SD)**	1.35 ± 0.18	1.37 ± 0.38	0.842	1.36 ± 0.22	1.28 ± 0.19	0.296
**DV S/D (mean ± SD)**	1.14 ± 0.13	1.28 ± 0.51	0.318	1.29 ± 0.26	1.18 ± 0.15	0.221
**DV v/D (mean ± SD)**	0.85 ± 0.12	0.92 ± 0.14	0.131	0.95 ± 0.19	0.92 ± 0.13	0.526
**DV S (mean ± SD)**	55.82 ± 17.09	63.85 ± 19.86	0.244	59.55 ± 28.69	57.14 ± 18.57	0.735
**DV D (mean ± SD)**	49.62 ± 16.64	55.78 ± 25.49	0.449	46.49 ± 18.18	49.33 ± 17.46	0.643
**DV a (mean ± SD)**	29.89 ± 13.29	29.56 ± 18.13	0.954	29.25 ± 9.54	31.84 ± 13.10	0.555
**DV v (mean ± SD)**	41.76 ± 12.83	49.80 ± 20.01	0.211	42.73 ± 13.99	45.15 ± 15.56	0.650
**DV TAmax (mean ± SD)**	45.52 ± 14.68	52.69 ± 19.20	0.257	47.35 ± 19.03	47.82 ± 15.76	0.933
**CPR (mean ± SD)**	1.67 ± 0.41	1.87 ± 0.46	0.222	1.55 ± 0.45	1.79 ± 0.47	0.147

CANO: composite adverse neonatal outcome; PI: pulsatility index; UA: umbilical artery; S/D: systolic/diastolic; MCA: middle cerebral artery; PSV: peak systolic velocity; DV: ductus venosus; PLI: preload index; PVIV: peak velocity index for veins; PIV: pulsatility index for veins; S: systolic peak velocity; D: diastolic peak velocity; a: peak velocity during atrial contraction; v: peak velocity during isovolumetric ventricular relaxation; TAmax: time-averaged maximum velocity; CPR: cerebroplacental ratio; SD: standard deviation.

## Data Availability

Data are not publicly available due to ethical restrictions. Further inquiries can be directed to the corresponding author.

## Abbreviations

The following abbreviations are used in this manuscript:

AC	Abdominal circumference
AGA	Appropriate for gestational age
AUC	Area under the curve
BMI	Body mass index
BPD	Biparietal diameter
CANO	Composite adverse neonatal outcome
CPR	Cerebroplacental ratio
CRL	Crown–rump length
DV	Ductus venosus
EFW	Estimated fetal weight
EO-FGR	Early-onset fetal growth restriction
FGR	Fetal growth restriction
FL	Femur length
HC	Head circumference
IBM	International Business Machines
ISUOG	International Society of Ultrasound in Obstetrics and Gynecology
LO-FGR	Late-onset fetal growth restriction
MCA	Middle cerebral artery
NICU	Neonatal intensive care unit
OR	Odds ratio
PI	Pulsatility index
PSV	Peak systolic velocity
ROC	Receiver operating characteristic
RDS	Respiratory distress syndrome
SD	Standard deviation
SPSS	Statistical Package for the Social Sciences
TAmax	Time-averaged maximum velocity
UA	Umbilical artery
UtA	Uterine artery
